# Correction to [Anti‐PL‐12 anti‐synthetase syndrome manifesting with multiple digital ischemia: Case report & review of the literature]

**DOI:** 10.1002/ccr3.9456

**Published:** 2024-09-17

[Doumeth SA, Petrinec E, Chaudhary H, Mattar M. Anti‐PL‐12 anti‐synthetasesyndrome manifesting with multiple digitalischemia: Case report & review of the literature. ClinCase Rep. 2024;12:e9408. doi:10.1002/ccr3.9408].

In the published article,“Anti‐PL‐12 anti‐synthetasesyndrome manifesting with multiple digitalischemia: Case report & review of the literature,” the corresponding author's name was formatted incorrectly. It should read as Abi Doumeth S.

We apologize for this error.

